# Host-microbiota-parasite interactions in two wild sparid fish species, *Diplodus annularis and Oblada melanura* (Teleostei, Sparidae) over a year: a pilot study

**DOI:** 10.1186/s12866-023-03086-3

**Published:** 2023-11-16

**Authors:** Mathilde Scheifler, Elodie Magnanou, Sophie Sanchez-Brosseau, Yves Desdevises

**Affiliations:** Sorbonne Université, CNRS, Biologie Intégrative Des Organismes Marins, BIOM, Banyuls-sur-Mer, F-66650 France

**Keywords:** Teleost fish, Parasitism, Abiotic factor, Immunity, Metabarcoding, Seasonal fluctuation

## Abstract

**Background:**

The microbiota in fish external mucus is mainly known for having a role in homeostasis and protection against pathogens, but recent evidence suggests it is also involved in the host-specificity of some ectoparasites. In this study, we investigated the influence of seasonality and environmental factors on both fish external microbiota and monogenean gill ectoparasites abundance and diversity and assessed the level of covariations between monogenean and bacterial communities across seasons. To do so, we assessed skin and gill microbiota of two sparid species, *Oblada melanura* and *Diplodus annularis*, over a year and collected their specific monogenean ectoparasites belonging to the *Lamellodiscus* genus.

**Results:**

Our results revealed that diversity and structure of skin and gill mucus microbiota were strongly affected by seasonality, mainly by the variations of temperature, with specific fish-associated bacterial taxa for each season. The diversity and abundance of parasites were also influenced by seasonality, with the abundance of some *Lamellodiscus* species significantly correlated to temperature. Numerous positive and negative correlations between the abundance of given bacterial genera and *Lamellodiscus* species were observed throughout the year, suggesting their differential interaction across seasons.

**Conclusions:**

The present study is one of the first to demonstrate the influence of seasonality and related abiotic factors on fish external microbiota over a year. We further identified potential interactions between gill microbiota and parasite occurrence in wild fish populations, improving current knowledge and understanding of the establishment of host-specificity.

**Supplementary Information:**

The online version contains supplementary material available at 10.1186/s12866-023-03086-3.

## Background

Interaction between species is one of the key determinants of both spatial and temporal dynamics of biological communities [1]. Teleost fish species are inhabited by a large array of symbiotic macro- and microorganisms that establish parasitic, commensal, or mutualistic interactions with their host [2–5]. Bacterial communities from teleost mucus have been shown to be of primary importance in numerous biological and ecological functions, such as intra- and interspecific chemical communication (i.e., social behavior, predation) or host fitness, by modulating the immune system or protection against pathogens [6–12]. Several studies showed that bacterial diversity and structure associated with external fish mucus are both tissue- and species-specific and are significantly different from the bacterial communities observed in the surrounding water [13–18].

In the past few years, progress has been made in describing the wide diversity of fish bacterial communities, but most of previous studies focused on fish gut microbiota, and on commercial fish species and/or model species, largely sidelining wild fish populations [19–22]. In addition, only a few studies have investigated variations of external fish mucus microbiota across time [23–25]. Therefore, most of our knowledge on fish external microbiota is based on a transient representation of these potentially dynamic communities (i.e., species collected at a given time or at short intervals) [26–28]. Some studies reported variability in bacterial composition in response to environmental shifts, such as temperature [13, 24], salinity [20] or pH [29]. Other studies highlighted that some host characteristics, such as modulation of the fish immune system (i.e., defense mechanisms), could also explain this variability [12, 30]. Understanding the determinants shaping complex fish microbiota requires knowing how these bacterial communities change over time and which abiotic and/or biotic factors explain the diversity and composition of external microbiota, particularly in wild fish populations.

In addition to being involved in homeostasis and immunity against pathogens, external fish mucus attracts and harbors specific parasitic species, such as monogeneans [2, 31]. Monogeneans (Platyhelminthes) are highly host-specific ectoparasites that are abundant on the fish skin and gills [32, 33]. Monogenean eggs are released in the water column, hatch into ciliated larvae (i.e., oncomiracidia) that are attracted to the mucus of fish [34, 35]. After reaching the host skin, larvae lose their ciliature and most of them migrate to the fish gills to develop in adults. External fish mucus microbiota has been hypothesized to be involved in the interaction with these ectoparasites [36]. Indeed, by producing attractive and/or repulsive chemical compounds, external bacterial communities appear to play a role in the mechanisms of establishment of monogenean specificity. Moreover, several studies highlighted that the presence of parasitic species, mainly endoparasites for now (e.g., digeneans, cestodes or nematodes), can act on and alter the composition of fish gut microbiota, which can affect host health and fitness [37–39]. Multiple negative and positive correlations between parasitic intensity and/or abundance and specific bacterial taxa have been reported in several fish species [40–42]. For example, Hennersdorf et al. (2016) showed that two potential bacterial pathogens, *Vibrio* sp. and *Photobacterium* sp., were negatively correlated with the total number of endoparasites in three Indonesian fish species (*Epinephelus fuscoguttatus*, *E. sexfasciatus* and *Atule mate*) [40]. However, in contrast to mammalian species or fish gut microbiota, almost nothing is known about the interaction dynamics between skin or gill mucus microbiota and ectoparasites, such as monogeneans [36], and how it changes across seasons.

In this study, we focused on a well-known fish-parasite interaction in the Mediterranean Sea: the association between Sparidae (Perciformes) and their specific monogenean gill ectoparasites belonging to the *Lamellodiscus* genus. We studied two sparid species, easy to sample throughout the year, *Oblada melanura* (saddled seabream) and *Diplodus annularis* (annular seabream) that respectively harbor 2 (*L. elegans* and *L. gracilis*) and 7 (*L. coronatus, L. elegans*, *L. ergensi, L. fraternus, L. furcosus, L. gracilis *and* L. ignoratus*) *Lamellodiscus* species [43–45]. The aim of this study is first to characterize the bacterial communities living within the external mucus (from skin and gills) of two wild fish species over one year and assess the effect of seasonality and environmental factors on the structure and diversity of these two microbiota. We also characterized the *Lamellodiscus* communities in each fish individual to investigate how gill mucus microbiota varies with ectoparasites composition and abundances across seasons.

## Materials and methods

### From samplings to 16S sequencing processing

Sample collection, DNA extraction, 16S rRNA sequencing and sequence processing were performed following the protocol of Scheifler et al. [18]. In short, *D. annularis* and *O. melanura* individuals were collected between August 2018 and May 2019 in the Bay of Banyuls-sur-Mer (northwest Mediterranean, France). For all individuals, skin mucus and gill mucus were collected using sterile spatula and scissors. Seawater was also collected during each season to compare bacterial communities from the water to those associated with fish tissues. DNA was extracted with the Quick-DNA Fecal/Soil Microbe MiniPrep Kit (Zymo Research, Orange, California). The V3-V4 region of the 16S rRNA genes was amplified by PCR using primers 341F and 805R [46, 47]. Amplicons were sequenced using Illumina 2 × 300 bp MiSeq sequencing (FASTERIS SA, Switzerland). Sequence analysis was performed using the QIIME2 software [48, 49]. Reads were denoised using DADA2 resulting in a list of Amplicon Sequence Variants (ASVs) [50]. Taxonomic affiliations were obtained using the SILVA 138 reference database [51, 52]. ASVs represented by a single sequence or matching with “Archaea”, “Eukaryota” and “Unassigned” were finally removed. The rarefaction analysis showed that two skin mucus and one gill mucus samples had lower sampling depth than the others (with 264, 386 and 449 reads). These three samples were discarded, and the data were rarefied to 13,900 sequences. Unfortunately, after this rarefaction, there was no skin mucus samples left for *O. melanura* during summer (Additional file 1: Table S1).

### Characterization of *Lamellodiscus *communities in fish gills

For each fish individual, seven gill arches were used to determine *Lamellodiscus* diversity and abundance. The haptor and male copulatory organ morphology were used to identify the species, under an optical microscope [43–45].

### Data and statistical analyses

All statistical analyses were performed using the rarefied ASV table. Bacterial alpha diversity was calculated using Shannon and Faith’s phylogenetic indices with the R package *phyloseq* [53]. A General Linear Model (GLM) was applied to identify which variables (fish species, tissue— skin and gill mucus and water communities —and season) influence bacterial diversity (*lme4* package). Differences between groups were then identified using estimated marginal means comparisons using the *emmeans* package. The effect of fish species, tissue and season, as well as the possible interactions between these factors on bacterial communities was assessed by Permutational Multivariate Analysis of Variance (PERMANOVA) and pairwise comparisons for both weighted Unifrac and Bray–Curtis indices (*adonis2* function, R package *vegan*). Considering that some bacteria from the surrounding environment can be found on the fish tissue by chance (e.g., caused by water currents), these analyses were also performed without considering sequences identified in water samples (i.e., on the “specific” gill and skin mucus microbiota). The number of shared ASVs among seasons was calculated and represented using a Venn diagram for skin and gill mucus for each fish species. Bacterial taxa (i.e., phylum, class, order, family and genus) contributing the most to the differences between skin and gill mucus bacterial communities across seasons were assessed with a Linear discriminant analysis Effect Size (LEfSe) and LDA scores [54]. To investigate the relationships between bacterial composition and environmental factors, we also performed Canonical Correspondence Analyses (CCA) with the R package *vegan*. The influence of environmental variables (temperature, salinity, oxygen O_2_, NH_4_, NO_3_, NO_2_, PO_4_, SiOH_4_, chlorophyll a and suspended particulate matter (Additional file 1: Table S2), measured by the SOMLIT (Service d’Observation en Milieu Littoral, https://www.somlit.fr/)) were tested on both skin and gill mucus bacterial communities with an ANOVA using the R package *vegan*.

ANOVA (or Kruskal–Wallis rank sum tests when data were not normally distributed) and Tukey post hoc tests (or post hoc Conover-Iman tests) were performed to analyze the influence of seasonality on abundance and Shannon diversity index [55] of *Lamellodiscus* species in each fish species. Associations between parasitic abundance (total abundance and abundance of each *Lamellodiscus* species) and temperature were quantified using negative binomial generalized linear models (MASS package in R). We performed negative binomial regression as negative binomial distribution of parasite load is commonly observed in nature [42, 56] and abundances of *Lamellodiscus* parasites were overdispersed (variance higher than the mean) in the present study. Correlations between parasitic Shannon diversity and gill mucus microbiota diversity (Faith’s and Shannon index) were computed and their significance assessed using Pearson’s correlation tests.

Mantel tests were performed to analyze associations between the composition and abundance of all *Lamellodiscus* species (based on a Euclidean matrix) and gill mucus bacterial communities (based on Bray–Curtis dissimilarities) for each fish species. To investigate the potential interactions between abundance of *Lamellodiscus* species and the composition of gill mucus bacterial communities across a year, we performed again negative binomial regressions for each fish species. A baseline negative binomial generalized linear model with only temperature was constructed (*Lamellodiscus* species ~ Temperature). A second model that included *Lamellodiscus* abundance and temperature was then constructed for each bacterial genus individually (*Lamellodiscus* species ~ Temperature + bacterial genus). Bacterial genera for which the second model was found to be significant (lowest AIC and p-value < 0.05) were kept. Only bacterial genera with an abundance > 0.05% in gill mucus microbiota and a prevalence of at least 10% in the samples were considered for downstream analysis. Again, these analyses were performed with and without considering sequences from water samples.

## Results

A total of around 3.8 million sequences assigned to bacteria (i.e., filtering out reads belonging to Archaea, Eukaryota and unassigned reads) was obtained across all samples. After sequences rarefaction, 11,550 ASVs were recovered from the gill mucus, skin mucus and water samples (45, 38 and 12 samples respectively). *Proteobacteria* was the most abundant phylum in gill mucus, skin mucus and water samples (67.1%, 48.2% and 60.4% respectively; Additional file 1: Table S3). The skin and gill mucus samples were also composed of *Firmicutes* (25.4% and 17.5% respectively), followed by *Bacteroidetes* (8.2% and 3.7%) and *Actinobacteria* (8.9% and 3.4%). On the contrary, *Bacteroidetes* was the second most abundant phylum in water samples (16.6%), followed by *Cyanobacteria* (8.2%) (Additional file 1: Table S3 and Figure S1).

### Bacterial diversity of skin and gill mucus is affected by seasonality

**Fig. 1:**
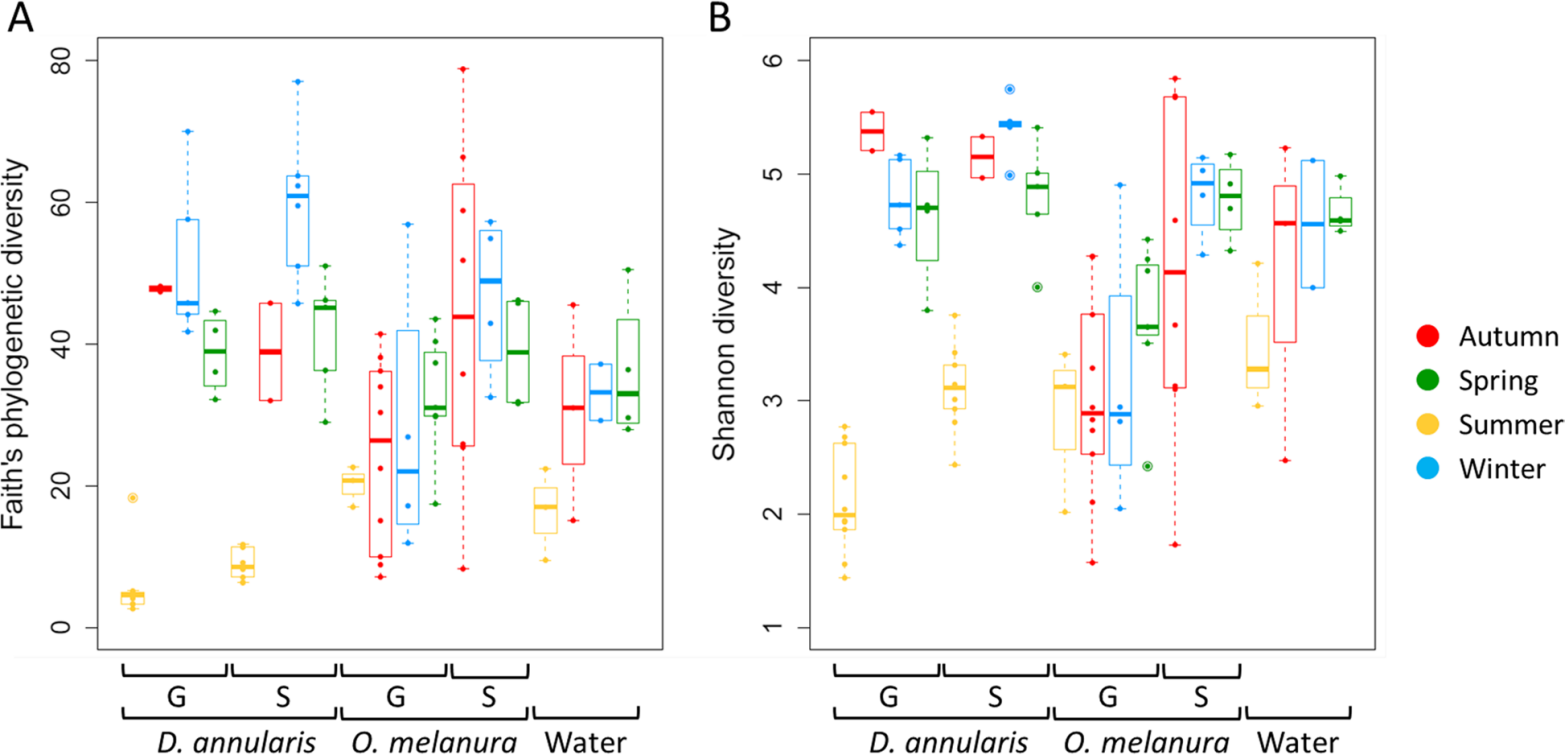
Faith’s phylogenetic **A** and Shannon **B** diversity of *Diplodus annularis*, *Oblada melanura* and water samples during each season. G, Gill mucus; S, Skin

We measured the diversity within bacterial communities (alpha diversity) using two indices: the Shannon diversity index reflecting taxonomic richness and evenness and the Faith’s phylogenetic index that considers the phylogenetic richness. For both Shannon and Faith diversity index, the GLM results suggested that bacterial communities from skin mucus, gill mucus and surrounding water were significantly different from each other’s (GLM, *p* < 0.001; Table 1). No significant difference in diversity between fish species was found (GLM, *p* > 0.05; Table 1). Moreover, for both alpha diversity metrics, a significant effect of seasonality was observed (GLM, *p* < 0.001). A significant interaction between seasonality and fish species was also observed (GLM, *p* < 0.01; Table 1), mostly explained by the low bacterial diversity in summer compared to other seasons in skin and gill mucus of *D. annularis* (Pairwise comparisons, *p* < 0.05, Fig. 1A-B).

**Table 1:** Results of the GLM analysis for bacterial diversity. Significant *p*-values are in bold

	*p*-value	
	Shannon	Faith
Tissue	** < 0.001**	** < 0.001**
Fish species	0.61	0.07
Season	** < 0.001**	** < 0.001**
Tissue:Fish species	0.22	0.10
Fish species:Season	** < 0.01**	** < 0.001**

After model simplification, the glm model used for statistical analyses was: glm(Diversity ~ Tissue + Fish species + Season + Tissue:Fish species + Fish species:Season)

### Seasonality acts on bacterial dissimilarities

**Fig. 2:**
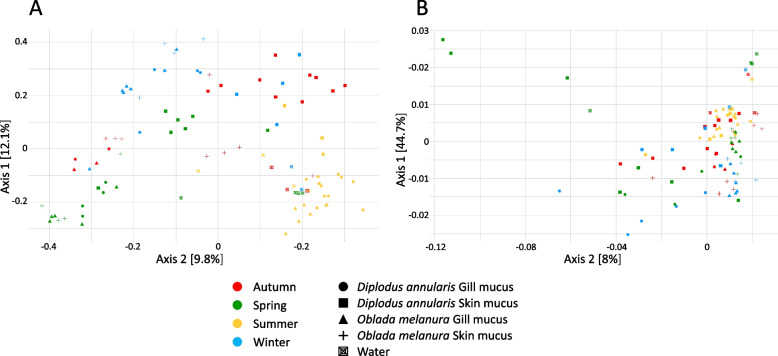
PCoA plots representing all fish gill mucus, skin mucus and water samples. PCoA based on Bray–Curtis **A** and weighted Unifrac **B** dissimilarity values. Each dot represents one community (water sample or fish individual). Color and shape of dots indicate season, fish species and tissue

To determine which factors explain the variability between and within skin mucus, gill mucus and water bacterial communities, two metrics were used: the Bray–Curtis dissimilarity index (BC), which takes into account the relative abundances of each ASV, and the weighted Unifrac distance (WU), that incorporates both relative abundance and phylogenetic relationships between ASVs. A principal coordinate analysis (PCoA) was used to plot both BC and WU distances (Fig. 2). Significant differences between bacterial communities from skin mucus, gill mucus and surrounding water were obtained (PERMANOVA of PCoA groups, for BC and WU respectively: *p* < 0.05, R^2^ = 0.11; *p* < 0.05, R^2^ = 0.13; Fig. 2). The PCoA plot based on Bray–Curtis values explained a low percentage of variability between communities (21.9%) compared to the PCoA based on WU distances (52.7%). Gill mucus, skin mucus and water communities harbored specific bacterial taxa (9.8%, 6% and 10.1% of ASVs respectively) (Additional file 1: Figure S2). These three compartments shared 23.7% of ASVs and the greatest compositional similarity was observed between gill and skin mucus (43.7% of ASVs) (Additional file 1: Figure S2).

**Fig. 3:**
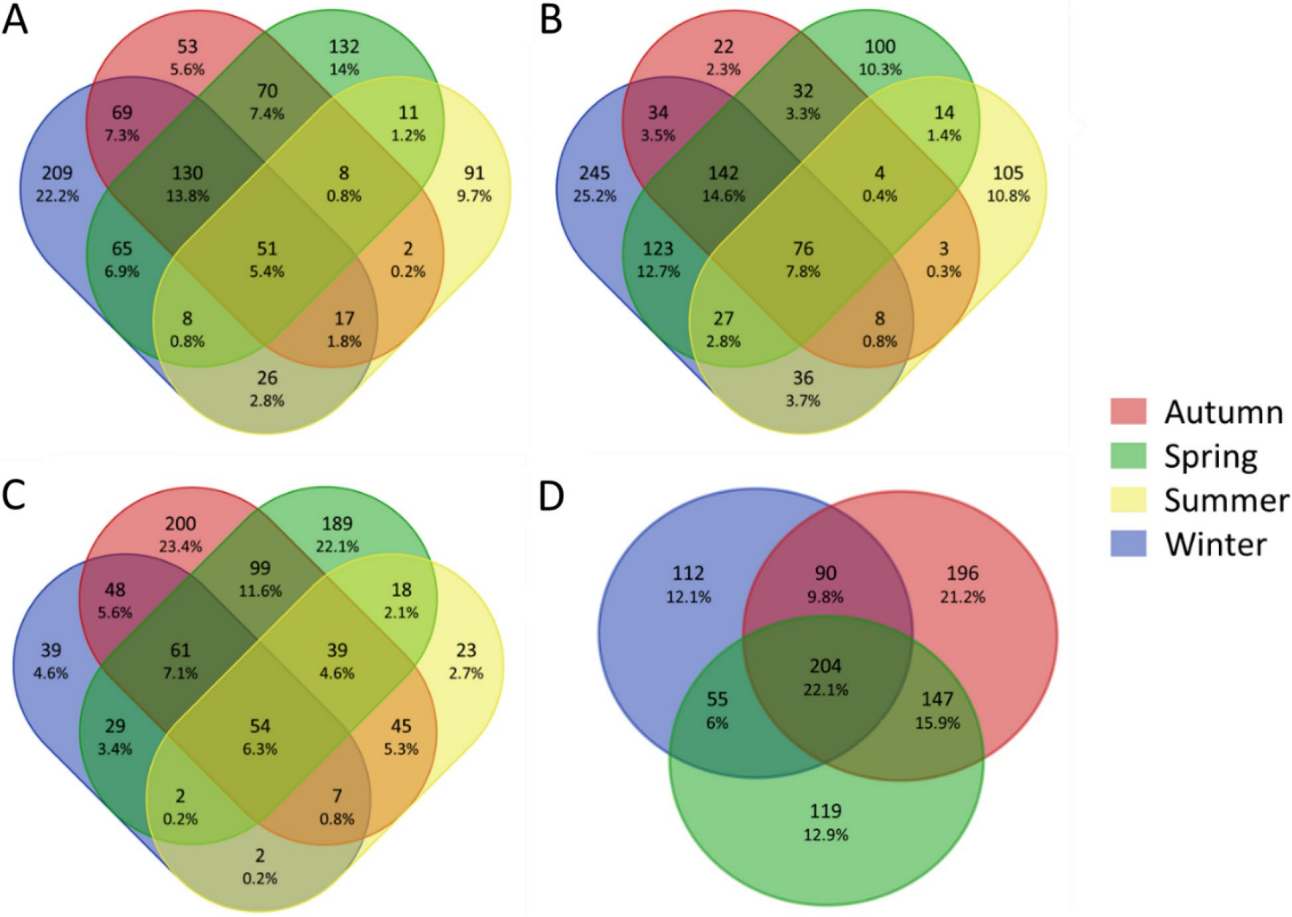
Venn diagrams representing percentages of shared ASVs among seasons with a 0.005% abundance cutoff. *Diplodus annularis *gill mucus **A**, skin mucus **B** and *Oblada melanura* gill mucus **C** and skin mucus **D**

We observed that seasonality explained variations in bacterial community composition among gill and skin mucus, with a stronger effect on skin microbiota compared to gills (PERMANOVA on BC and WU distances, *p* < 0.05, R^2^ = 0.21 and R^2^ = 0.14 respectively). For both metrics, the analyses also revealed significant interactions between seasonality and fish species and between seasonality and tissue (*p* < 0.05). The effect of seasonality was thus different according to the fish tissue and fish species. The same significant results were obtained without considering sequences from water samples, with a small decrease in influence of the seasonality on gill and skin mucus microbiota dissimilarities (PERMANOVA on BC and WU distances, *p* < 0.05, R^2^ = 0.15 and R^2^ = 0.11 respectively). Moreover, numerous shared bacterial taxa were found between seasons: around 5.9% of ASVs were shared within gill mucus microbiota during the year (5.4% for *D. annularis*, 6.3% for *O. melanura*) while around 15% of ASVs were shared within skin mucus microbiota (7.8% for *D. annularis*, 22.1% for *O. melanura*, the latter percentage being based only on 3 seasons) (Fig. 3). Gill mucus and skin mucus also harbored a huge number of specific ASVs depending on the season (Fig. 3). For example, *D. annularis* gill mucus microbiota displayed 25.2% of specific ASVs during winter (Fig. 3).

Furthermore, the same bacterial taxa displayed higher abundances in both gill and skin mucus microbiota depending on seasons (Lefse analysis; Table 2). For example, gill and skin mucus microbiota of both fish species harbored higher abundances of *Vibrio* and *Aliivibrio* in winter, *Bacteroidetes*, *Verrucomicrobia*, *Flavobacteriales*, *Planococcus* or *Gramella* in spring and *Bacillales* and *Enterobacteriaceae* in summer (Table 2). Skin and gill mucus microbiota were also differentially affected by seasonality. For example, the abundances of the two genera *Photobacterium* and *Shewanella* were higher in autumn compared to other seasons in gill mucus, whereas the skin mucus was mostly colonized by *Tumebacillus* and *Rubritalea* during this same season (Table 2). Within the same tissue, the two fish species were also affected differently by seasonality (Additional file 1: Table S4). For example, in autumn, *Illumatobacter*, *Halioglobus* or *Pseudahrensia* abundances were significantly more important in the gill mucus of *D. annularis*, whereas *Photobacterium* and *Shewanella* abundances were higher in *O. melanura* gill mucus (Additional file 1: Table S4).

**Table 2:** Differences in bacterial abundances between seasons within skin and gill mucus microbiota. LDA scores were calculated using Linear discriminant analysis Effect Size (LEfSe). Only bacterial taxa that raised an LDA score > 2 were included. Bacterial taxa significantly enriched during the same season for both skin and gill mucus microbiota are in bold

Skin mucus	LDA score	Enriched in…	Gill mucus	LDA score	Enriched in…
*Phylum*					
**Actinobacteria**	2.96	Winter	**Actinobacteria**	2.53	Winter
**Bacteroidetes**	3.15	Spring	**Bacteroidetes**	2.76	Spring
Firmicutes	3.37	Summer	Fusobacteria	2.21	Winter
Planctomycetes	2.51	Winter	Planctomycetes	2.24	Spring
**Verrucomicrobia**	2.45	Spring	**Verrucomicrobia**	2.53	Spring
*Class*					
**Acidimicrobiia**	2.84	Winter	**Acidimicrobiia**	2.37	Winter
Actinobacteria	2.24	Spring	**Alphaproteobacteria**	3.08	Spring
**Alphaproteobacteria**	3.27	Spring	**Bacteroidia**	2.76	Spring
Bacilli	3.40	Summer	Fusobacteriia	2.21	Winter
**Bacteroidia**	3.14	Spring	Planctomycetacia	2.24	Spring
Clostridia	2.24	Winter	**Verrucomicrobiae**	2.53	Spring
Deltaproteobacteria	2.35	Winter			
Gammaproteobacteria	3.38	Summer			
Planctomycetacia	2.51	Winter			
**Verrucomicrobiae**	2.45	Spring			
*Order*					
Alteromonadales	2.06	Autumn	Alteromonadales	2.50	Summer
**Bacillales**	3.34	Summer	**Bacillales**	3.28	Summer
Bradymonadales	2.05	Spring	Betaproteobacteriales	2.69	Spring
Clostridiales	2.24	Winter	**Enterobacteriales**	3.12	Summer
**Enterobacteriales**	3.22	Summer	**Flavobacteriales**	2.76	Spring
**Flavobacteriales**	3.13	Spring	**Microtrichales**	2.32	Winter
Lactobacillales	2.49	Summer	Pirellulales	2.21	Spring
**Microtrichales**	2.81	Winter	**Rhizobiales**	2.59	Winter
Pirellulales	2.29	Winter	**Rhodobacterales**	2.74	Spring
Planctomycetales	2.10	Winter	**Sphingomonadales**	2.13	Spring
Propionibacteriales	2.18	Spring	**Verrucomicrobiales**	2.53	Spring
Pseudomonadales	3.14	Autumn	Vibrionales	3.41	Autumn
**Rhizobiales**	2.69	Winter			
**Rhodobacterales**	3.06	Spring			
**Sphingomonadales**	2.58	Spring			
**Verrucomicrobiales**	2.44	Spring			
Vibrionales	2.99	Winter			
*Family*					
Alicyclobacillaceae	2.05	Autumn	Betaproteobacteriales IS	2.55	Spring
Bacillaceae	3.02	Winter	Carnobacteriaceae	2.29	Autumn
**Enterobacteriaceae**	3.22	Summer	**Enterobacteriaceae**	3.12	Summer
Family_XII	3.35	Summer	**Flavobacteriaceae**	2.75	Spring
**Flavobacteriaceae**	3.12	Spring	Moraxellaceae	2.20	Spring
Ilumatobacteraceae	2.30	Winter	Pirellulaceae	2.20	Spring
Nocardioidaceae	2.18	Spring	**Planococcaceae**	2.59	Spring
Pirellulaceae	2.22	Winter	**Rhizobiaceae**	2.32	Winter
**Planococcaceae**	2.96	Spring	**Rhodobacteraceae**	2.74	Spring
Pseudomonadaceae	2.40	Autumn	**Rubritaleaceae**	2.47	Spring
**Rhizobiaceae**	2.41	Winter	Shewanellaceae	2.46	Autumn
**Rhodobacteraceae**	3.06	Spring	**Sphingomonadaceae**	2.13	Spring
**Rubritaleaceae**	2.36	Spring	Staphylococcaceae	3.24	Summer
**Sphingomonadaceae**	2.58	Spring	Vibrionaceae	3.41	Autumn
Vibrionaceae	2.99	Winter			
*Genus*					
***Aliivibrio***	2.22	Winter	***Aliivibrio***	2.92	Winter
*Bacillus*	3.02	Winter	*Cetobacterium*	2.07	Winter
*Citrobacter*	2.91	Summer	***Enterobacter***	3.10	Summer
***Enterobacter***	2.77	Summer	***Gramella***	2.36	Spring
*Erythrobacter*	2.17	Spring	***Paracoccus***	2.09	Spring
*Exiguobacterium*	3.35	Summer	*Photobacterium*	3.30	Autumn
***Gramella***	2.67	Spring	***Planococcus***	2.58	Spring
*Ilumatobacter*	2.30	Winter	*Psychrobacter*	2.16	Spring
*Nocardioides*	2.14	Spring	*Rubritalea*	2.27	Spring
***Paracoccus***	2.37	Spring	*Shewanella*	2.46	Autumn
***Planococcus***	2.95	Spring	*Staphylococcus*	3.24	Summer
*Rubritalea*	2.17	Autumn	***Sulfitobacter***	2.15	Spring
***Sulfitobacter***	2.64	Spring	***Vibrio***	3.03	Winter
*Tumebacillus*	2.05	Autumn			
***Vibrio***	2.47	Winter			
*Winogradskyella*	2.01	Spring			

**Fig. 4:**
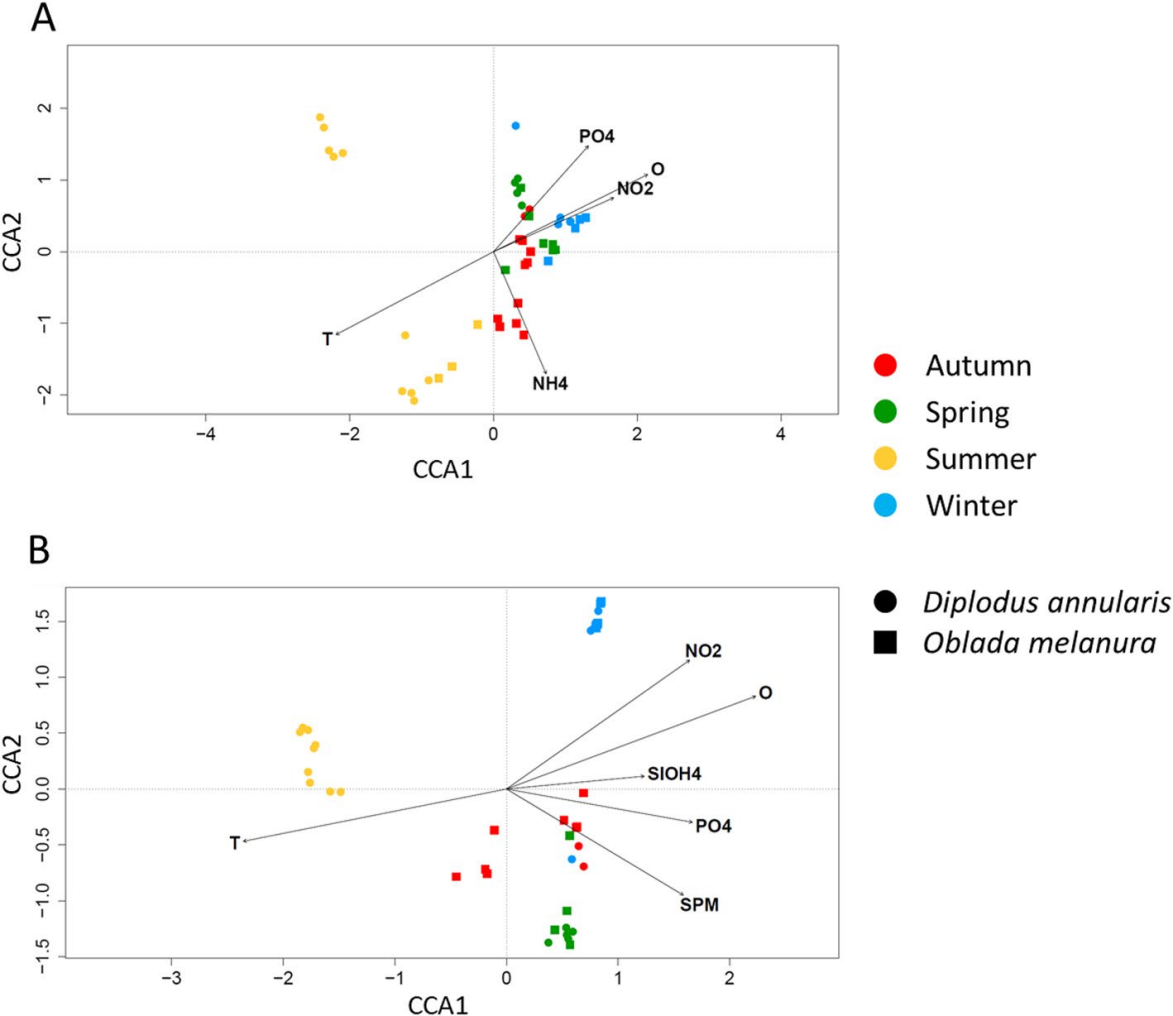
Canonical correspondence analysis of gills **A** and skin **B** microbiota in relation to environmental factors. Each dot represents one community. Color and shape of dots indicate season and fish species. T: Temperature; O: Oxygen; SPM: suspended particulate matter

Canonical correspondence analyses (CCA) were performed on the bacterial composition of gill and skin mucus to investigate the relationships between gill and skin bacterial composition and environmental factors. Consistent with the other analyses, both gill and skin microbiota showed a seasonal pattern (Fig. 4). The model, composed of the environmental explanatory factors, explained 24.9% and 34.1% (constrained inertia) of the total variance of gill and skin mucus microbiota composition respectively. Skin mucus microbiota seemed to be more influenced by seasonality and environment than gill mucus microbiota. These communities generally clustered according to the season but showed more divergence on the CCA plots during summer for gill mucus. The main explanatory factors were temperature (T) for both gill mucus and skin mucus (Fig. 4). Gill mucus composition was further influenced by 4 other variables: NH_4_, O_2_, NO_2_ and PO_4_ (*p* < 0.05). Concerning skin mucus composition, it was mainly influenced by oxygen, NO_2_, SiOH4, PO_4_ and suspended particulate matter (SPM) (Fig. 4).

### Effect of seasonality on *Lamellodiscus *diversity and abundance

**Fig. 5:**
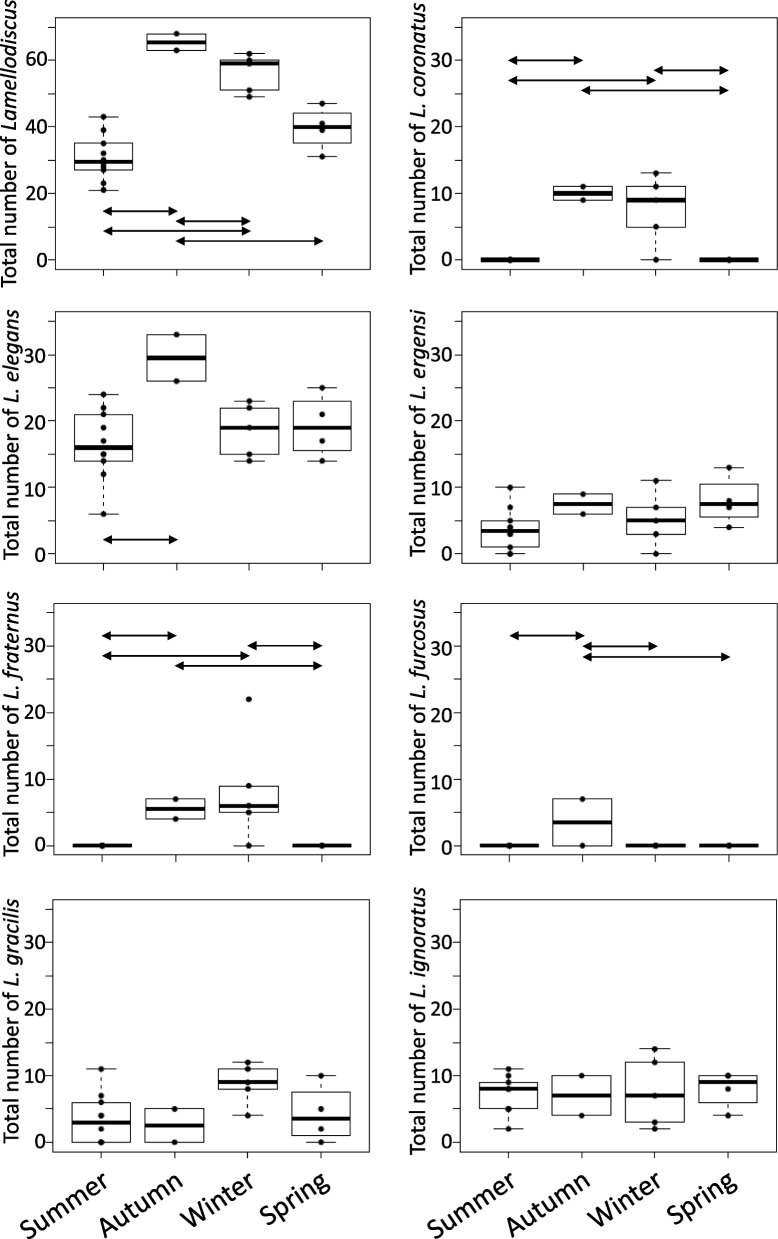
Abundance of all *Lamellodiscus* species in *Diplodus annularis *gill arches during each season. Arrows on plots represent significant differences between seasons (based on Tukey or Conover-Iman tests)

**Fig. 6:**
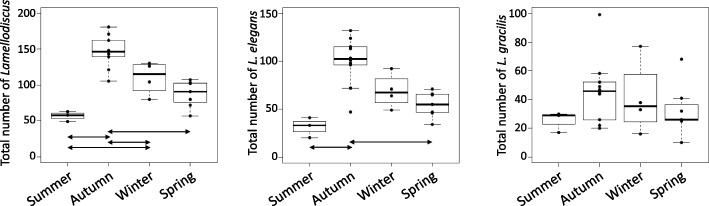
Abundance of all *Lamellodiscus *species in *Oblada melanura *gill arches during each season. Arrows on plots represent significant differences between seasons (based on Tukey or Conover-Iman tests)

Two and 7 *Lamellodicus* species were found in the gills of *O. melanura* and *D. annularis* (Additional file 1: Table S5) respectively. Specific richness of *Lamellodiscus* varied across seasons in *D. annularis*, while *L. elegans* and *L. gracilis* were both found during each season in *O. melanura* (Additional file 1: Table S5). The total abundance of *Lamellodiscus* was influenced by seasonality for both *D. annularis* and *O. melanura* (ANOVA, *p* < 0.05), with higher abundances in autumn and winter compared to other seasons (*p* < 0.05; Figs. 5 and 6). More specifically, differences of total abundance between seasons for both species are due to *L. elegans* abundances, which are also influenced by seasonality (ANOVA, *p* < 0.05) and are higher in autumn (*p* < 0.05; Figs. 5 and 6). Seasonality also affected the abundance of *L. coronatus, L. fraternus* and *L. furcosus* in *D.s annularis* (Fig. 5). Four *Lamellodiscus* species*, L. elegans*, *L. ignoratus*, *L. gracilis* and *L. ergensi* were present during each season, whereas *L. fraternus*, *L. furcosus* and *L. coronatus* were not found in spring and summer (Fig. 5). We also found similar abundances of *L. gracilis* across seasons for both fish species (Figs. 5 and 6). Moreover, significant negative correlations between total abundance (*r* = -0.65), *L. fraternus* (*r* = -0.58) and *L. coronatus* (*r* = -0.57) abundances and temperature were found (negative binomial regression, *p* < 0.05), suggesting that the increase in temperature led to a decrease of the abundances of these *Lamellodiscus* species. Finally, Shannon diversity for parasites displayed significant differences according to seasons in *D. annularis* with in general a higher diversity in autumn and winter (Fig. 5A; Additional file 1: Figure S3A and Table S5), whereas no influence was observed for *O. melanura* (Additional file 1: Figure S3B).

### Correlations between *Lamellodiscus* abundance and bacterial communities

First, both Faith’s phylogenetic and Shannon diversity indexes associated with gill mucus microbiota were positively correlated with parasite’s Shannon diversity only in *D. annularis* (Pearson correlation test, *p* < 0.05, *r* = 0.69 and *r* = 0.62 respectively), which suggests that an increase in *Lamellodiscus* diversity is linked to an increase in gill microbiota diversity in this fish species. Moreover, the composition and abundance of *Lamellodiscus* species were significantly correlated with the specific bacterial composition of fish gill mucus microbiota (i.e., without considering sequences from water samples) for both fish species (Mantel test, *p* < 0.05; *r* = 0.30 and *r* = 0.17 for *D. annularis* and *O. melanura*, respectively).

To elucidate potential correlations between *Lamellodiscus* species abundance and gill mucus microbiota, we performed negative binomial regressions and compared two models, one considering only temperature (*Lamellodiscus* species~Temperature) and another considering both temperature and genus abundance (*Lamellodiscus* species~Temperature+bacterial genus). We kept only bacterial genera for which the second model is the best-fit model. Numerous significant associations between bacterial genera and parasite composition and abundances were found (Table 3). After removing water sequences (and associated ASVs) from gill mucus microbiota (i.e., specific gill mucus microbiota), we identified 6 and 26 correlations for *O. melanura* and *D. annularis* respectively, among which 12 were positive (i.e., indicating that the abundance of a bacterial genus is positively linked with *Lamellodiscus* species abundance) and 20 were negative. Among others, the abundance of *L. coronatus* in *D. annularis* was correlated with 8 specific bacterial taxa: 3 negative associations with *Exiguobacterium*, *Maribacter* and *Neorickettsia* and 5 positive associations with *Ilumatobacter*, *Rhodopirellula, Roseibacillus, Rubritalea and Tumebacillus* (Table 3). We noticed that the abundance of both *L. coronatus* and *L. fraternus*, present in *D. annularis* was linked to 5 bacterial genera (*Ilumatobacter*, *Maribacter*, *Neorickettsia*, *Rubritalea*, *Tumebacillus*), with generally similar positive or negative correlation coefficients (Table 3). *L. elegans* and *L. gracilis* abundances displayed correlations with the abundance of different bacterial genera depending on the fish host species. For example, in *O. melanura*, the abundance of *L. elegans* was correlated with *Bacillus*, *Cyanobium, Photobacterium*, *Staphylococcus* and *Stenotrophomonas*, whereas these associations were all absent in *D. annularis*. For *L. gracilis*, there was a correlation with *Pseudahrensia* in *O. melanura*, but with *Enterovibrio* in *D. annularis* (Table 3).

By considering the entire gill mucus microbiota (i.e., with water sequences), the composition and abundance of *Lamellodiscus* species is still correlated to the variation of gill mucus microbiota for both fish species (Mantel test, *p* < 0.05; *r* = 0.34 and *r* = 0.17 for *D. annularis* and *O. melanura*, respectively). Using negative binomial regressions, more significant correlations were identified between the abundance of *Lamellodiscus* species and bacterial genera (Table 3). A proportion of 65.6% of the significant correlations identified in the first place (i.e., without water sequences) were also found in this second analysis.

**Table 3:**
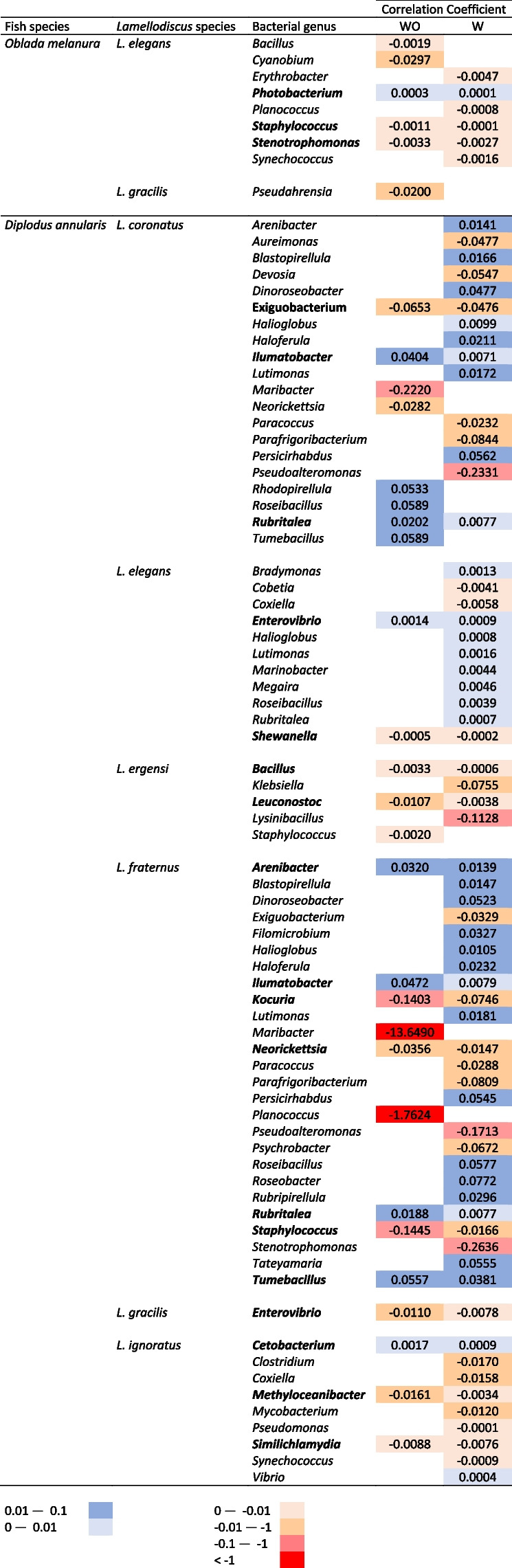
Coefficients from negative binomial generalized models between the abundance of *Lamellodiscus* species and bacterial genera. These coefficients were computed with (W) without (WO) considering sequences in water samples. Bacterial genera present in the two categories are in bold. The color of each cell represents the direction of the slope (red is negative, blue is positive)

## Discussion

### External fish microbiota is driven by both biotic and abiotic factors

Bacterial communities from skin mucus, gill mucus and environmental water differ significantly from each other and across time, both in terms of diversity and composition. Despite their constant exposition to water bacterial communities, skin and gill mucus do not harbor the same assemblages than the surrounding water. Indeed, these two tissues harbor different bacterial abundances both from each other and from the surrounding environment (Additional file 1: Table S3 and Figures S1 and S2). As previously reported in the same fish family (Sparidae), skin mucus harbored more *Firmicutes* than gill mucus which presents higher abundances in *Proteobacteria* [17, 18]. We also found that external microbiota (i.e., both skin and gill microbiota) responds differently to seasonal variations according to the fish species. *D. annularis* and *O. melanura* share the same global environment in the Mediterranean Sea but are characterized by different ecological traits. For example, *O. melanura* is an omnivorous fish whereas *D. annularis* is carnivorous [57]. Two studies on coral reef fish and sparids have shown that fish diet partly explain dissimilarities in external fish microbiota [15, 18]. As seasonality can also influence availability of food and subsequently fish diet and/or food intake [58], we can hypothesize that diet plays a role in the variations of the external mucus microbiota. Similarly, other fish ecological factors, such as its position in the water column or its behavior, may also be influenced by season, and are then potential factors responsible of microbiota’s variations.

The diversity and the composition of both skin and gill mucus microbiota are linked to seasonality. Temperature was one of the most important abiotic factors structuring bacterial communities, followed by O_2_, NH_4_ (for gills communities) and NO_2_ (for skin mucus). Several studies have already suggested that seasonality and associated changes in environmental factors could strongly influence fish microbiota diversity and structure. Indeed, huge bacterial changes have been observed after acclimation to salinity and in response to temperature, pH or oxygen fluctuations, for both fish internal (i.e., gut [19, 24, 59–66]) and external microbiota (i.e., skin [23–25]). Our results need, however, to be confirmed on a larger scale, by conducting more fish samplings per season. Moreover, we also highlighted in this study that the variability of skin and gill mucus microbiota was also explained by the seasonality when we take into account sequences from water samples. The composition of the microbiota seems to be, at least in part, determined by the bacterial communities present in the surrounding water, as suggested in several studies (both skin and gut microbiota, [67, 68]). Indeed, seawater bacterial communities exhibit clear temporal shifts in densities, diversity and composition according to seasons [69]. The variation of biotic and abiotic factors of the surrounding water, such as temperature, salinity, nutrients availability, phytoplankton blooms, eutrophication or concentration of pollutants and toxins, seem to drive the composition of water bacterial communities. Reoccurring patterns of bacterial composition have also been observed across years [70–72]. Bacteria are also known to require specific optimum environmental conditions to grow and environmental shifts, especially temperature and nutrients availability, can influence this growth [73] and subsequently affect gene expression, as well as the structure and function of bacterial communities [74]. These environmental changes can thus induce rapid community changes, which can affect colonization and competition processes between bacterial taxa [75].

### Influence of the fish immune system on external microbiota

The environment in which an animal lives can affect its physiology, including its immune system. Abiotic environmental factors, such as temperature, salinity or oxygen, have been reported to influence fish immune system [76–78]. Several studies highlighted an upregulation of immunity responses associated to the increase of water temperature in various fish species, such as turbot (*Scophthalmus maximus* [79]) and ayu (*Pecoglossus altivelis* [80]). Huang et al. (2011) pointed out that an increase in water temperature induced an increase in lysozyme concentration, a common innate immune enzyme involved in protection against gram-positive bacteria [79], and Immunoglobulin-M (IgM), the most important class of antibodies in fish species [81]. Sugahara & Eguchi (2012) demonstrated that the increase of water temperature induced a better protection against pathogens from the *Flavobacterium* genus [80]. It has been also suggested that low temperatures could lead to a decreased ability of fish to respond to pathogens (i.e., a deletion of adaptive immunity), affecting their health and increasing subsequently the potential risk of infection by pathogenic bacteria [82]. In the present study, we observed higher abundances of the genera *Vibrio* and *Allivibrio* in winter (in both skin and gill mucus), where some strains are known to be pathogenic [83, 84]. We can hypothesize that these higher abundances of *Vibrio* and *Allivibrio* have a link with the fish immune system. However, a study of the immune system of fish across seasons (e.g., fish condition factor, IgM levels) must be carried out to confirm this hypothesis. In the same way, several studies showed that increases in photoperiod, salinity, oxygen, or pH result in a general increase in immune functions [85] (for a complete review see [86, 87]). Therefore, it is possible that seasonal changes affecting skin and gill mucus microbiota are due to the influence of several factors, including abiotic factors linked to the water composition (e.g., concentration of oxygen, salinity,…), but also the bacterial diversity and composition of surrounding water and fish immune system.

### Seasonality influences on the putative relationship between parasites and microbiota

Two and 7 *Lamellodiscus* species were found in *O. melanura* and *D. annularis* respectively. This pattern of presence/absence of *Lamellodiscus* species observed within these two fish species is in accordance with previous studies [43–45]. The total abundance of *Lamellodiscus* individuals displayed clear and significant differences between seasons, with generally higher abundance in autumn and winter compared to spring and summer for both fish species. Fluctuations of *Lamellodiscus* abundances across seasons have already been observed in several sparid species, such as *Sparus aurata*, *Diplodus puntazzo* or *Pagellus erythrinus* [88–90]. In this study, some species were observed across all seasons, such as *L. elegans*, with fluctuating abundance between seasons (e.g., higher in autumn), whereas other species were only reported in one or two seasons, such as *L. coronatus* and *L. fraternus*. This disparity of parasite species occurrence across seasons highlights fluctuations regarding the life cycle of parasites.

Previous studies have already suggested that seasonality and associated abiotic fluctuations are important factors driving the occurrence and abundance of monogeneans by directly acting upon egg hatching, development, reproduction or survival [91–94]. Water temperature is considered to be the most important factor influencing the timing of monogeneans life cycle [95, 96]. Generally, high water temperatures (in spring and summer) promote reproduction rate, faster hatching of monogenean eggs and subsequently larval spread to fish hosts [95, 97, 98]. However, the effect of temperature differs among monogenean species. For example, in the *Dactylogyrus* genus (*Lamellodiscus* belong to the same order Dactylogyridea), some species prefer low temperatures (*D. lamellatus*, *D. extensus*) whereas others prefer higher temperatures (*D. vastator*, *D. ctenopharyngodonis*) [99, 100], suggesting that each species is adapted to specific environmental conditions. The influence of salinity on the prevalence and intensity of monogenean infection has also been reported [94, 101–103]. *Paeonodus lagunaris,* a parasite of redbreast tilapia *Coptodon rendalli*, displayed higher prevalence and intensity in summer when water salinity is the lowest [101]. In addition, it has been suggested that environmental factors, such as temperature or salinity, influence monogenean host-specificity across seasons [104]. Indeed, *Gyrodactylus salaris* showed morphological variations of its opisthohaptoral sclerified parts between seasons, which can alter its definitive establishment on fish gills [105–107].

In this study, we examined the associations between the presence and abundance of different ectoparasitic monogenean species and the composition of gill mucus microbiota in two fish species over a year. We found that 2 and 6 *Lamellodiscus* species, in *O. melanura* and *D. annularis* respectively, were positively or negatively linked to the abundance of specific bacterial genera (Table 3). To our knowledge, this is the first time that correlations between monogenean abundances and gill mucus microbiota composition are observed on a seasonal basis. We pointed out that some associations seem to be species-specific (i.e., the abundance of a given *Lamellodiscus* species over the year is linked to specific bacterial genera), while other *Lamellodiscus* species, such as *L. coronatus* and *L. fraternus*, displayed positive or negative correlations with same bacterial genera. The present observations support the hypothesis that external fish mucus microbiota plays a role in the interaction with pathogens. Recent studies proposed that these mechanisms of repulsion and attraction between parasites and host external mucus involve molecules, which are produced, at least partly, by bacteria or directly by parasites [31, 36, 108, 109]. Environmental factors, especially temperature, can strongly influence immune defense in fish, as well as their physiology, and then may indirectly affect monogenean occurrence and abundance [110]. For example, Rohlenová et al. (2011) showed correlations between seasonality, host immunity and physiology (e.g., fish condition factor, steroid hormones, IgM circulating levels, hematological parameters) and the intensity of monogenean infection in the fish *Cyprinus carpio* [110]. Indeed, if seasonal variations induce shifts in the diversity and bacterial composition of external mucus, the production of metabolites (involved in attraction and/or protection toward parasites) by these communities will change (negatively or positively, depending on the fish health status), which may potentially influence the interactions between fish external mucus and parasites.

## Conclusions

The present study is among the first to assess the impact on seasonal variations and environmental factors on skin and gill mucus microbiota in wild fish species. We highlighted that skin and gill mucus microbiota harbored specific bacterial communities at each season both in terms of diversity and composition for two fish species. These results support the hypothesis that both the surrounding water and host-related factors influence host colonization by bacterial taxa. In addition, numerous significant associations between the abundance of gill mucus bacteria and *Lamellodiscus* species were found across a year, suggesting a functional association between these two biological compartments.

### Supplementary Information

**Additional file 1: Table S1. **Total number of DNA samples used in this study, after rarefaction. **Table S2.** Abiotic descriptors for each sampling day. Data were obtained from the station SOLA, located in the Bay of Banyuls-sur-Mer, close to the fish sampling site. **Table S3.** Percentage of the most abundant phyla found in gill mucus, skin mucus and water samples. **Table S4. **Differences in bacterial abundances between fish species and seasons within skin and gill microbiota. LDA scores were calculated using Linear discriminant analysis Effect Size (LEfSe). Only bacterial taxa that raised an LDA score >2 were included. Bacterial taxa significantly enriched during the same season, in the same species and for both skin and gill mucus microbiota are in bold. Dann: *Diplodus annularis; *Omel:* Oblada melanura. ***Table S5. **Abundance of each *Lamellodiscus *species within the gills of each fish individual for each season. A: Autumn; Sp: Spring; Su: Summer; W: Winter. **Figure S1.** Relative abundances of bacterial phyla within gill and skin mucus during each season. A, Autumn; W, Winter; Sp, Spring; Su, Summer. **Figure S2.** Venn diagramm representing shared ASVs between skin, gill mucus and water samples. Based on a 0.005% abundance cutoff*. ***Figure S3. **Shannon’s diversity of *Lamellodiscus *species in *Diplodus annularis *(A) and *Oblada melanura *(B) gill arches during each season. Arrows represent significant differences between seasons (based on Tukey tests, *p*-value < 0.05).

## Data Availability

Sequence data will be available upon publication in the NCBI Sequence Read Archive (SRA, https://www.ncbi.nlm.nih.gov/sra) database belonging to the BioProject PRJNA748412.

## Abbreviations

ASV: Amplicon Sequence Variant
CCA: Canonical Correspondence Analysis
GLM: General Linear Model
LDA: Linear Discriminant Analysis
LEfSe: Linear discriminant analysis Effect Size
PCoA: Principal Coordinates Analysis
PERMANOVA: Permutational Multivariate Analysis of Variance
BC: Bray-Curtis dissimilarity index
WU: Weighted Unifrac dissimilarity index
