# Supplementary material for: Host-microbiota-parasite interactions in two wild sparid fish species, Diplodus annularis and Oblada melanura (Teleostei, Sparidae) over a year: a pilot study
Source: BMC Microbiol. 2023 Nov 16;23:340. doi: 10.1186/s12866-023-03086-3 (PMC10652623; doi:10.1186/s12866-023-03086-3)
Supplement: Supplementary file 1 — Additional file 1: Table S1. Total number of DNA samples used in this study, after rarefaction. Table S2. Abiotic descriptors for each sampling day. Data were obtained from the station SOLA, located in the Bay of Banyuls-sur-Mer, close to the fish sampling site. Table S3. Percentage of the most abundant phyla found in gill mucus, skin mucus and water samples. Table S4. Differences in bacterial abundances between fish species and seasons within skin and gill microbiota. LDA scores were calculated using Linear discriminant analysis Effect Size (LEfSe). Only bacterial taxa that raised an LDA score >2 were included. Bacterial taxa significantly enriched during the same season, in the same species and for both skin and gill mucus microbiota are in bold. Dann: Diplodus annularis; Omel: Oblada melanura. Table S5. Abundance of each Lamellodiscus species within the gills of each fish individual for each season. A: Autumn; Sp: Spring; Su: Summer; W: Winter. Figure S1. Relative abundances of bacterial phyla within gill and skin mucus during each season. A, Autumn; W, Winter; Sp, Spring; Su, Summer. Figure S2. Venn diagramm representing shared ASVs between skin, gill mucus and water samples. Based on a 0.005% abundance cutoff. Figure S3. Shannon’s diversity of Lamellodiscus species in Diplodus annularis (A) and Oblada melanura (B) gill arches during each season. Arrows represent significant differences between seasons (based on Tukey tests, p-value < 0.05). [file 12866_2023_3086_MOESM1_ESM.docx]

Table S1. Total number of DNA samples used in this study, after rarefaction.

|  | Summer | | Autumn | | Winter | | Spring | |
| --- | --- | --- | --- | --- | --- | --- | --- | --- |
|  | S | G | S | G | S | G | S | G |
| *Diplodus annularis* | 9^3,4^ | 10^3,4^ | 2^7^ | 2^7^ | 6^8,9^ | 5^8,9^ | 5^12,13^ | 4^12,13^ |
| *Oblada melanura* | 0 | 3^3^ | 8^6,7^ | 10^6,7^ | 4^9^ | 4^9^ | 4^11-13^ | 7^11-13^ |
| Water | 3^1-3^ | | 3^5-7^ | | 2^8,9^ | | 4^10-13^ | |

S, Skin mucus; G, Gill mucus

In 2018: ^1^June 26; ^2^July 13; ^3^August 29; ^4^September 4; ^5^September 28; ^6^October 5; ^7^December 4

In 2019: ^8^February 8; ^9^March 6; ^10^March 22; ^11^March 28; ^12^May 10; ^13^May 21

Table S2. Abiotic descriptors for each sampling day. Data were obtained from the station SOLA, located in the Bay of Banyuls-sur-Mer, close to the fish sampling site.

|  | 26/06/2018 | 13/07/2018 | 29/08/2018 | 04/09/2018 | 28/09/2018 | 05/10/2018 | 04/12/2018 | 08/02/2019 | 06/03/2018 | 22/03/2019 | 28/03/2019 | 10/05/2019 | 21/05/2019 |
| --- | --- | --- | --- | --- | --- | --- | --- | --- | --- | --- | --- | --- | --- |
| Temperature (T) (ºC) | 20.40 | 22.10 | 21.56 | 20.93 | 21.56 | 18.76 | 15.87 | 10.68 | 12.50 | 13.10 | 12.56 | 14.51 | 14.92 |
| Salinity (S) (PSU) | 37.42 | 38.16 | 38.22 | 38.10 | 38.02 | 38.17 | 37.48 | 37.816 | 37.78 | 38.02 | 38.15 | 38.04 | 38.14 |
| Oxygen (O) (mL/L) | 5.27 | 5.17 | 5.11 | 5.11 | 5.07 | 5.25 | 5.64 | 6.12 | 6.18 | 5.84 | 5.95 | 5.85 | 5.77 |
| NH_4_ (µmoL/L) | 0.02 | 0.04 | 0.06 | 0.01 | 0.06 | 0.07 | 0.06 | 0.04 | 0.03 | 0.04 | 0.09 | 0.03 | 0.02 |
| NO_3_ (µmoL/L) | 1.26 | 0.02 | 0.02 | 0.06 | 0.02 | 0.02 | 1.48 | 1.12 | 1.84 | 1.20 | 0.7 | 0.05 | 0.13 |
| NO_2_ (µmoL/L) | 0.16 | 0.01 | 0.01 | 0.01 | 0.01 | 0.01 | 0.21 | 0.36 | 0.21 | 0.18 | 0.27 | 0.02 | 0.02 |
| PO_4_ (µmoL/L) | 0.03 | 0.01 | 0.01 | 0.02 | 0.02 | 0.01 | 0.03 | 0.07 | 0.03 | 0.02 | 0.05 | 0.04 | 0.03 |
| SiOH_4_ (µmoL/L) | 0.77 | 1.31 | 0.91 | 1.19 | 0.97 | 1.18 | 4.85 | 1.45 | 2.09 | 1.91 | 2.29 | 1.07 | 1.33 |
| Suspended particulate matter (SPM) (mg/L) | 1.62 | 1.73 | 0.32 | 0.41 | 1.14 | 0.53 | 0.69 | 2.21 | 0.82 | 1.66 | 1.77 | 1.41 | 1.22 |
| Chlorophylle a (mg/L) (CHLA) | 1.53 | 1.53 | 0.24 | 0.29 | 0.32 | 0.35 | 0.81 | 1.1 | 0.77 | 0.61 | 0.62 | 1.1 | 0.59 |

Table S3. Percentage of the most abundant phyla found in gill mucus, skin mucus and water samples.

| Phylum | Gill mucus | Skin mucus | Water |
| --- | --- | --- | --- |
| Actinobacteria | 3.4 | 8.9 | 3.7 |
| Bacteroidetes | 3.7 | 8.2 | 16.6 |
| Chloroflexi | 0.3 | 0.7 | 0.2 |
| Cyanobacteria | 1.1 | 1.0 | 8.2 |
| Firmicutes | 17.5 | 25.4 | 5.0 |
| Fusobacteria | 1.5 | 0.3 | 0.0 |
| Planctomycetes | 1.9 | 3.1 | 1.5 |
| Proteobacteria | 67.1 | 48.2 | 60.4 |
| Verrucomicrobia | 2.3 | 2.9 | 2.3 |
| Others | 1.2 | 1.4 | 2.0 |

Table S4. Differences in bacterial abundances between fish species and seasons within skin and gill microbiota. LDA scores were calculated using Linear discriminant analysis Effect Size (LEfSe). Only bacterial taxa that raised an LDA score >2 were included. Bacterial taxa significantly enriched during the same season, in the same species and for both skin and gill mucus microbiota are in bold. Dann: *Diplodus annularis;* Omel: *Oblada melanura*

| Gills mucus | LDA score | Enriched in… |  | Skin mucus | LDA score | Enriched in… |
| --- | --- | --- | --- | --- | --- | --- |
| *Phylum* |  |  |  |  |  |  |
| Actinobacteria | 2.75 | Dann - Winter |  | Actinobacteria | 2.97 | Dann - Winter |
| Bacteroidetes | 3.12 | Dann - Autumn |  | Bacteroidetes | 3.21 | Dann - Spring |
| Cyanobacteria | 2.3 | Omel - Summer |  | Chloroflexi | 2.02 | Dann - Winter |
| Fusobacteria | 2.33 | Dann - Winter |  | Firmicutes | 3.45 | Dann - Summer |
| Planctomycetes | 2.53 | Dann - Autumn |  | Planctomycetes | 2.59 | Dann - Winter |
| Proteobacteria | 3.46 | Omel - Winter |  | Verrucomicrobia | 2.68 | Dann - Autumn |
| Verrucomicrobia | 2.78 | Dann - Autumn |  |  |  |  |
| *Class* |  |  |  |  |  |  |
| Acidimicrobiia | 2.6 | Dann - Autumn |  | Acidimicrobiia | 2.84 | Dann - Winter |
| Actinobacteria | 2.29 | Dann - Spring |  | Alphaproteobacteria | 3.31 | Omel - Spring |
| Alphaproteobacteria | 3.17 | Dann - Autumn |  | Bacilli | 3.47 | Dann - Summer |
| Bacilli | 3.46 | Omel - Summer |  | Bacteroidia | 3.2 | Dann - Spring |
| Bacteroidia | 3.11 | Dann - Autumn |  | Clostridia | 2.41 | Omel - Winter |
| Deltaproteobacteria | 2.29 | Dann - Autumn |  | Deltaproteobacteria | 2.42 | Dann - Autumn |
| Fusobacteriia | 2.33 | Dann - Winter |  | Gammaproteobacteria | 3.4 | Dann - Summer |
| Gammaproteobacteria | 3.63 | Omel - Winter |  | Planctomycetacia | 2.58 | Dann - Winter |
| Oxyphotobacteria | 2.3 | Omel - Summer |  | Verrucomicrobiae | 2.68 | Dann - Autumn |
| Planctomycetacia | 2.52 | Dann - Autumn |  |  |  |  |
| Verrucomicrobiae | 2.78 | Dann - Autumn |  |  |  |  |
| *Order* |  |  |  |  |  |  |
| Alteromonadales | 2.61 | Dann - Summer |  | Alteromonadales | 2.14 | Omel - Autumn |
| Bacillales | 3.47 | Omel - Summer |  | Bacillales | 3.43 | Dann - Summer |
| Betaproteobacteriales | 2.83 | Omel - Spring |  | Bradymonadales | 2.12 | Omel - Spring |
| Bradymonadales | 2.19 | Dann - Autumn |  | Cellvibrionales | 2.32 | Dann - Autumn |
| Cellvibrionales | 2.5 | Dann - Autumn |  | Clostridiales | 2.41 | Omel - Winter |
| Enterobacteriales | 3.24 | Dann - Summer |  | Enterobacteriales | 3.22 | Dann - Summer |
| Flavobacteriales | 3.1 | Dann - Autumn |  | Flavobacteriales | 3.19 | Dann - Spring |
| Fusobacteriales | 2.33 | Dann - Winter |  | Microtrichales | 2.83 | Omel - Winter |
| Microtrichales | 2.55 | Dann - Autumn |  | Pirellulales | 2.36 | Dann - Winter |
| Pirellulales | 2.46 | Dann - Autumn |  | Planctomycetales | 2.18 | Dann - Winter |
| Propionibacteriales | 2.11 | Dann - Spring |  | Propionibacteriales | 2.21 | Omel - Spring |
| Rhizobiales | 2.81 | Dann - Winter |  | Rhizobiales | 2.7 | Omel - Winter |
| Rhodobacterales | 2.92 | Dann - Autumn |  | Rhodobacterales | 3.08 | Omel - Spring |
| Sphingomonadales | 2.43 | Dann - Spring |  | K70 group | 2.12 | Dann - Autumn |
| Synechococcales | 2.3 | Omel - Summer |  | Sphingomonadales | 2.6 | Omel - Spring |
| Verrucomicrobiales | 2.78 | Dann - Autumn |  | Verrucomicrobiales | 2.68 | Dann - Autumn |
| Vibrionales | 3.7 | Omel - Winter |  | Vibrionales | 3.27 | Omel - Winter |
| *Family* |  |  |  |  |  |  |
| Betaproteobacteriales IS | 2.66 | Omel - Spring |  | Alicyclobacillaceae | 2.1 | Omel - Autumn |
| Bradymonadaceae | 2.18 | Dann - Autumn |  | Bacillaceae | 3.03 | Omel - Winter |
| Burkholderiaceae | 2.46 | Dann - Winter |  | Bradymonadaceae | 2.02 | Omel - Spring |
| Carnobacteriaceae | 2.36 | Omel - Autumn |  | Carnobacteriaceae | 2.09 | Dann - Winter |
| Cyanobiaceae | 2.3 | Omel - Summer |  | Clostridiaceae 1 | 2.27 | Omel - Winter |
| DEV007 | 2.04 | Dann - Autumn |  | DEV007 | 2.01 | Dann - Autumn |
| Enterobacteriaceae | 3.24 | Dann - Summer |  | Enterobacteriaceae | 3.22 | Dann - Summer |
| Family XII | 2.61 | Dann - Summer |  | Family XII | 3.35 | Dann - Summer |
| Flavobacteriaceae | 3.1 | Dann - Autumn |  | Flavobacteriaceae | 3.18 | Dann - Spring |
| Halieaceae | 2.44 | Dann - Autumn |  | Halieaceae | 2.28 | Dann - Autumn |
| Ilumatobacteraceae | 2.4 | Dann - Autumn |  | Ilumatobacteraceae | 2.33 | Dann - Autumn |
| Moraxellaceae | 2.45 | Dann - Spring |  | Nocardioidaceae | 2.21 | Omel - Spring |
| Nocardioidaceae | 2.1 | Dann - Spring |  | Pirellulaceae | 2.33 | Dann - Autumn |
| Pirellulaceae | 2.44 | Dann - Autumn |  | Planococcaceae | 2.97 | Omel - Spring |
| Planococcaceae | 2.85 | Dann - Spring |  | Pseudoalteromonadaceae | 2.01 | Omel - Autumn |
| Pseudomonadaceae | 3.16 | Omel - Spring |  | Rhizobiaceae | 2.41 | Omel - Winter |
| Rhizobiaceae | 2.56 | Dann - Winter |  | Rhodobacteraceae | 3.08 | Omel - Spring |
| Rhodobacteraceae | 2.92 | Dann - Autumn |  | Rubritaleaceae | 2.59 | Omel - Spring |
| Rubritaleaceae | 2.69 | Dann - Autumn |  | Sphingomonadaceae | 2.6 | Omel - Spring |
| Shewanellaceae | 2.56 | Omel - Autumn |  | Vibrionaceae | 3.27 | Omel - Winter |
| Sphingomonadaceae | 2.43 | Dann - Spring |  |  |  |  |
| Staphylococcaceae | 3.46 | Omel - Summer |  |  |  |  |
| Vibrionaceae | 3.7 | Omel - Winter |  |  |  |  |
| *Genus* |  |  |  |  |  |  |
| *Aliivibrio* | 3.24 | Omel - Winter |  | *Aliivibrio* | 2.59 | Omel - Winter |
| *Arenibacter* | 2.04 | Dann - Autumn |  | *Bacillus* | 3.03 | Omel - Winter |
| *Blastopirellula* | 2.04 | Dann - Autumn |  | *Blastopirellula* | 2.04 | Dann - Autumn |
| *Bradymonas* | 2.16 | Dann - Autumn |  | *Citrobacter* | 2.91 | Dann - Summer |
| *Delftia* | 2.27 | Omel - Spring |  | *Clostridium* | 2.09 | Omel - Winter |
| *Enterobacter* | 3.22 | Dann - Summer |  | *Enterobacter* | 2.77 | Dann - Summer |
| *Erythrobacter* | 2.07 | Dann - Spring |  | *Erythrobacter* | 2.2 | Omel - Spring |
| *Exiguobacterium* | 2.61 | Dann - Summer |  | *Exiguobacterium* | 3.35 | Dann - Summer |
| *Gramella* | 2.65 | Dann - Spring |  | *Gramella* | 2.85 | Dann - Autumn |
| *Halioglobus* | 2.35 | Dann - Autumn |  | *Halioglobus* | 2.21 | Dann - Autumn |
| *Ilumatobacter* | 2.4 | Dann - Autumn |  | *Ilumatobacter* | 2.33 | Dann - Autumn |
| *Lutimonas* | 2.13 | Dann - Autumn |  | *Lutimonas* | 2.04 | Dann - Autumn |
| *Mesonia* | 2.16 | Dann - Spring |  | *Maribacter* | 2.04 | Dann - Spring |
| *Nocardioides* | 2.05 | Dann - Spring |  | *Mesonia* | 2.15 | Dann - Spring |
| *Paracoccus* | 2.33 | Dann - Spring |  | *Nocardioides* | 2.18 | Omel - Spring |
| *Photobacterium* | 3.46 | Omel - Autumn |  | *Notassigned* | 3.23 | Dann - Autumn |
| *Planococcus* | 2.83 | Dann - Spring |  | *Paracoccus* | 2.38 | Omel - Spring |
| *Pseudahrensia* | 2.16 | Dann - Autumn |  | *Photobacterium* | 2.97 | Omel - Winter |
| *Pseudomonas* | 3.16 | Omel - Spring |  | *Planococcus* | 2.96 | Omel - Spring |
| *Psychrobacter* | 2.44 | Dann - Spring |  | *Pseudahrensia* | 2.17 | Dann - Autumn |
| *Rubritalea* | 2.49 | Dann - Autumn |  | *Pseudoalteromonas* | 2.01 | Omel - Autumn |
| *Shewanella* | 2.56 | Omel - Autumn |  | *Rubritalea* | 2.43 | Omel - Spring |
| *Staphylococcus* | 3.46 | Omel - Summer |  | *Ruegeria* | 2.06 | Dann - Autumn |
| *Sulfitobacter* | 2.38 | Dann - Autumn |  | *Sulfitobacter* | 2.64 | Omel - Spring |
| *Synechococcus* | 2.2 | Dann - Spring |  | *Trichococcus* | 2.09 | Dann - Winter |
| *Vibrio* | 3.34 | Omel - Winter |  | *Tumebacillus* | 2.1 | Omel - Autumn |
|  |  |  |  | *Vibrio* | 2.75 | Omel - Winter |
|  |  |  |  | *Winogradskyella* | 2.13 | Dann - Spring |

Table S5. Abundance of each *Lamellodiscus* species within the gills of each fish individual for each season. A: Autumn; Sp: Spring; Su: Summer; W: Winter

| Fish species | Season | Total | *L. coronatus* | *L. elegans* | *L. ergensi* | *L. fraternus* | *L. furcosus* | *L. gracilis* | *L. ignoratus* | Specific richness |
| --- | --- | --- | --- | --- | --- | --- | --- | --- | --- | --- |
| *Oblada melanura* | A | 139 |  | 113 |  |  |  | 26 |  | 2 |
|  | A | 146 |  | 124 |  |  |  | 22 |  | 2 |
|  | A | 162 |  | 115 |  |  |  | 47 |  | 2 |
|  | A | 147 |  | 103 |  |  |  | 44 |  | 2 |
|  | A | 121 |  | 101 |  |  |  | 20 |  | 2 |
|  | A | 148 |  | 96 |  |  |  | 52 |  | 2 |
|  | A | 171 |  | 72 |  |  |  | 99 |  | 2 |
|  | A | 181 |  | 132 |  |  |  | 49 |  | 2 |
|  | A | 105 |  | 47 |  |  |  | 58 |  | 2 |
|  | A | 143 |  | 98 |  |  |  | 45 |  | 2 |
|  | W | 130 |  | 92 |  |  |  | 38 |  | 2 |
|  | W | 104 |  | 71 |  |  |  | 33 |  | 2 |
|  | W | 80 |  | 64 |  |  |  | 16 |  | 2 |
|  | W | 126 |  | 49 |  |  |  | 77 |  | 2 |
|  | Sp | 57 |  | 47 |  |  |  | 10 |  | 2 |
|  | Sp | 80 |  | 55 |  |  |  | 25 |  | 2 |
|  | Sp | 107 |  | 66 |  |  |  | 41 |  | 2 |
|  | Sp | 103 |  | 71 |  |  |  | 32 |  | 2 |
|  | Sp | 72 |  | 46 |  |  |  | 26 |  | 2 |
|  | Sp | 102 |  | 34 |  |  |  | 68 |  | 2 |
|  | Sp | 91 |  | 65 |  |  |  | 26 |  | 2 |
|  | Su | 58 |  | 41 |  |  |  | 17 |  | 2 |
|  | Su | 63 |  | 33 |  |  |  | 30 |  | 2 |
|  | Su | 49 |  | 20 |  |  |  | 29 |  | 2 |
| *Diplodus annularis* | A | 63 | 9 | 33 | 6 | 4 | 7 |  | 4 | 6 |
|  | A | 68 | 11 | 26 | 9 | 7 |  | 5 | 10 | 6 |
|  | W | 59 | 13 | 23 |  | 5 |  | 11 | 7 | 5 |
|  | W | 51 | 11 | 22 | 7 |  |  | 9 | 2 | 5 |
|  | W | 62 | 5 | 15 | 11 | 9 |  | 8 | 14 | 6 |
|  | W | 60 |  | 19 | 3 | 22 |  | 4 | 12 | 5 |
|  | W | 49 | 9 | 14 | 5 | 6 |  | 12 | 3 | 6 |
|  | Sp | 47 |  | 25 | 4 |  |  | 10 | 8 | 4 |
|  | Sp | 41 |  | 21 | 8 |  |  | 2 | 10 | 4 |
|  | Sp | 39 |  | 17 | 7 |  |  | 5 | 10 | 4 |
|  | Sp | 31 |  | 14 | 13 |  |  |  | 4 | 3 |
|  | Su | 32 |  | 15 | 1 |  |  | 6 | 10 | 4 |
|  | Su | 28 |  | 19 | 4 |  |  |  | 5 | 3 |
|  | Su | 27 |  | 6 | 10 |  |  | 2 | 9 | 4 |
|  | Su | 30 |  | 15 |  |  |  | 7 | 8 | 3 |
|  | Su | 23 |  | 14 |  |  |  | 4 | 5 | 3 |
|  | Su | 35 |  | 22 | 5 |  |  |  | 8 | 3 |
|  | Su | 21 |  | 12 | 4 |  |  |  | 5 | 3 |
|  | Su | 39 |  | 21 | 3 |  |  | 4 | 11 | 4 |
|  | Su | 29 |  | 24 | 3 |  |  |  | 2 | 3 |
|  | Su | 43 |  | 17 | 7 |  |  | 11 | 8 | 4 |


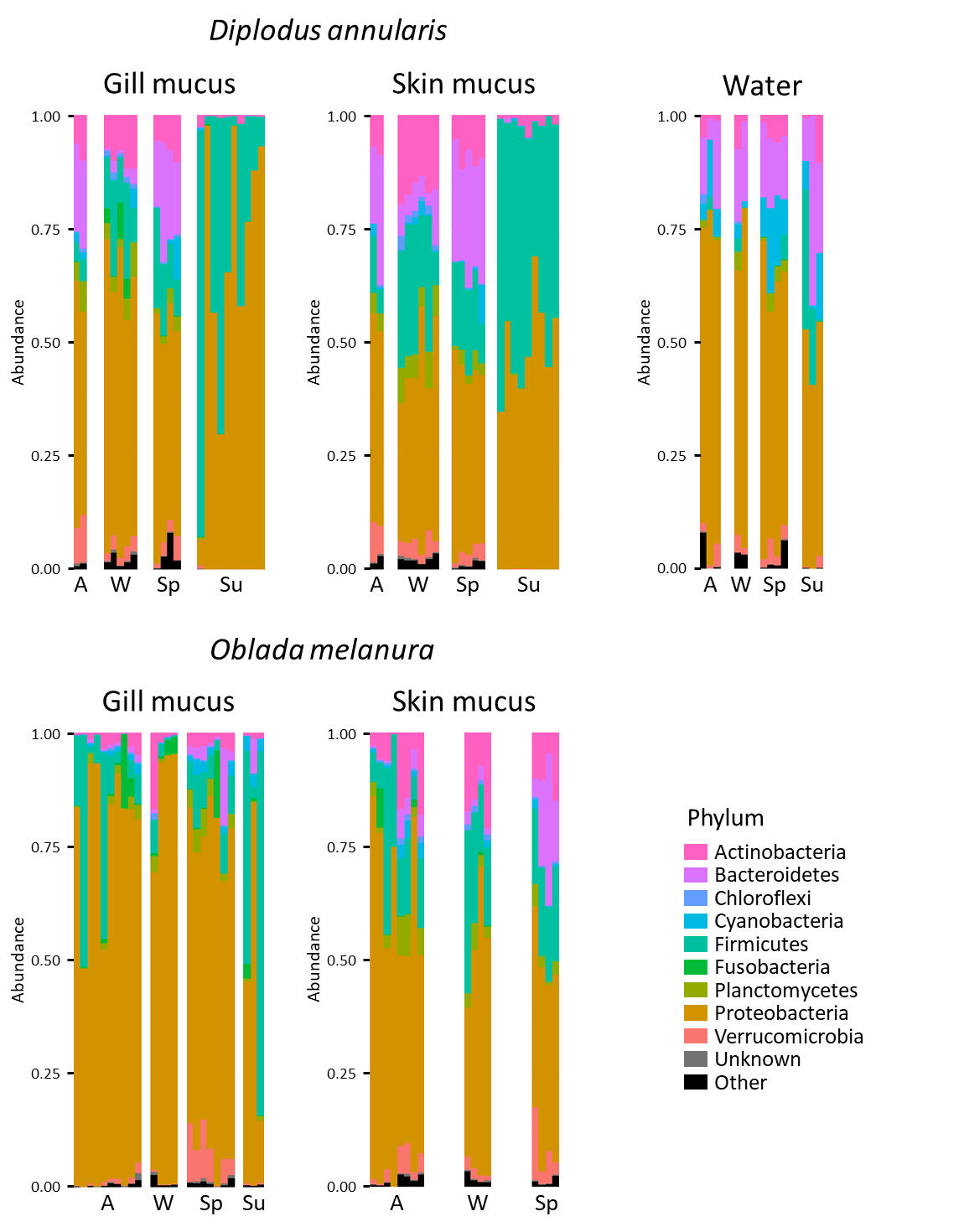


Figure S1. Relative abundances of bacterial phyla within gill and skin mucus during each season. A, Autumn; W, Winter; Sp, Spring; Su, Summer


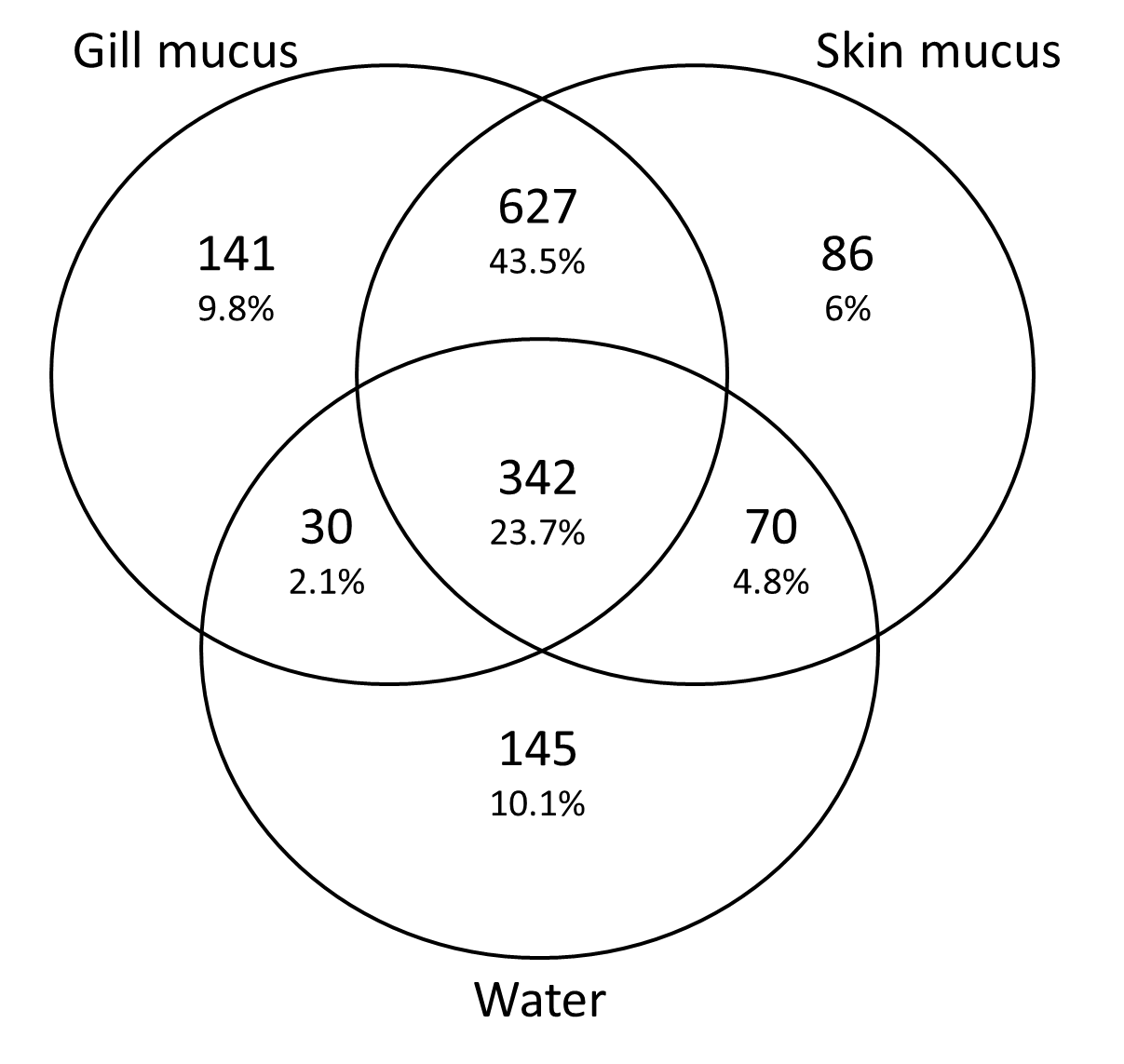


Figure S2. Venn diagramm representing shared ASVs between skin, gill mucus and water samples. Based on a 0.005% abundance cutoff*.*


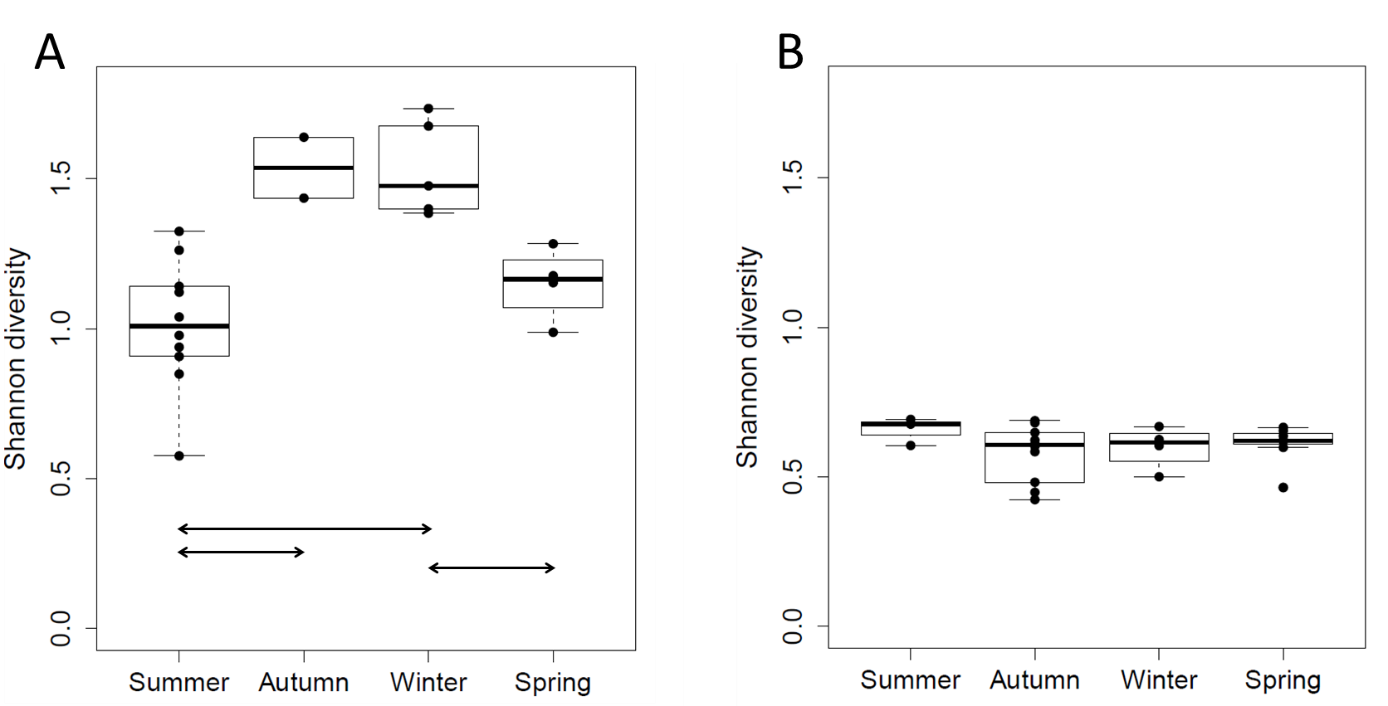


**Figure S3. Shannon’s diversity of Lamellodiscus species in Diplodus annularis (A) and Oblada melanura (B) gill arches during each season.** Arrows represent significant differences between seasons (based on Tukey tests, p-value < 0.05).
